# GABA Increases Electrical Excitability in a Subset of Human Unmyelinated Peripheral Axons

**DOI:** 10.1371/journal.pone.0008780

**Published:** 2010-01-20

**Authors:** Richard W. Carr, Ruth Sittl, Johannes Fleckenstein, Peter Grafe

**Affiliations:** 1 Institute of Physiology, Ludwig-Maximilians University, Munich, Germany; 2 Department of Anaesthesiology, Ludwig-Maximilians University, Munich, Germany; The Research Center of Neurobiology-Neurophysiology of Marseille, France

## Abstract

**Background:**

A proportion of small diameter primary sensory neurones innervating human skin are chemosensitive. They respond in a receptor dependent manner to chemical mediators of inflammation as well as naturally occurring algogens, thermogens and pruritogens. The neurotransmitter GABA is interesting in this respect because in animal models of neuropathic pain GABA pre-synaptically regulates nociceptive input to the spinal cord. However, the effect of GABA on human peripheral unmyelinated axons has not been established.

**Methodology/Principal Findings:**

Electrical stimulation was used to assess the effect of GABA on the electrical excitability of unmyelinated axons in isolated fascicles of human sural nerve. GABA (0.1–100 µM) increased electrical excitability in a subset (ca. 40%) of C-fibres in human sural nerve fascicles suggesting that axonal GABA sensitivity is selectively restricted to a sub-population of human unmyelinated axons. The effects of GABA were mediated by GABA_A_ receptors, being mimicked by bath application of the GABA_A_ agonist muscimol (0.1–30 µM) while the GABA_B_ agonist baclofen (10–30 µM) was without effect. Increases in excitability produced by GABA (10–30 µM) were blocked by the GABA_A_ antagonists gabazine (10–20 µM), bicuculline (10–20 µM) and picrotoxin (10–20 µM).

**Conclusions/Significance:**

Functional GABA_A_ receptors are present on a subset of unmyelinated primary afferents in humans and their activation depolarizes these axons, an effect likely due to an elevated intra-axonal chloride concentration. GABA_A_ receptor modulation may therefore regulate segmental and peripheral components of nociception.

## Introduction

Gamma-aminobutyric acid (GABA) is the predominant inhibitory neurotransmitter in the mammalian central nervous system. In addition to its role at synapses, GABA can also exert effects extra-synaptically via GABA_A_ receptors [1]–[3]. Adult primary afferent sensory neurons provide an interesting example in this respect. The cell bodies of dorsal root ganglion (DRG) neurones, which are devoid of synaptic contact, express functional GABA_A_ receptors (human: [4], [5], rat: [6], [7], cat: [8], rabbit: [9], chick: [10]). Functional GABA_A_ receptor mediated responses in the somata of DRG neurones is also evident in their unmyelinated axons (rat: [11]) where application of GABA results in depolarization (for review see [12], [13]). The GABA_A_ mediated depolarization is attributed to an elevated intracellular concentration of chloride in dorsal root ganglion neurones [14], a condition established by the predominance of NKCC1-mediated chloride uptake [15] over KCC2-mediated extrusion [16].

For neurones in the central nervous system, extra-synaptic axonal GABA_A_ receptors are often composed of sub-units with a high sensitivity to GABA allowing ambient concentrations of GABA to modulate neuronal excitability [2]. Similarly, in peripheral nerve, extra-synaptic axonal GABA_A_ receptors can modulate excitability and may be involved in GABAergic signalling between axons and neighbouring cells such as dermal fibroblasts [17] and subtypes of peripheral glia [18], [19]. Peripheral glia can take up GABA [20] and reversal of the GABA transporter [21] may result in release of the amino acid.

The effect of GABA on peripheral axons is dependent upon the intracellular chloride concentration which is reported to change following peripheral nerve injury in dorsal spinal horn neurones [22] as well as DRG neurones [23]. Changes in intercellular chloride concentration will inevitably alter the effects of GABA on primary sensory afferents both within the spinal cord as well as in the periphery and such changes may contribute to symptoms of neuropathic pain [24]. The presence of GABA_A_ receptors on the unmyelinated axons of primary sensory neurones is central to this concept. However it has not been established whether the axons of human primary sensory neurones express functional GABA_A_ receptors. The chemosensitivity of unmyelinated axons can be examined by tracking the electrical threshold of the compound C-fibre action potential generated in short isolated segments of isolated sural nerve [25] and in the present study this method has been used to characterize the effect of GABA on unmyelinated axons from humans.

## Results

Experiments were carried out on 53 isolated nerve fascicles from 16 human sural nerve segments.

### GABA increases C-fibre axonal excitability via GABA_A_ receptors

**Figure 1:**
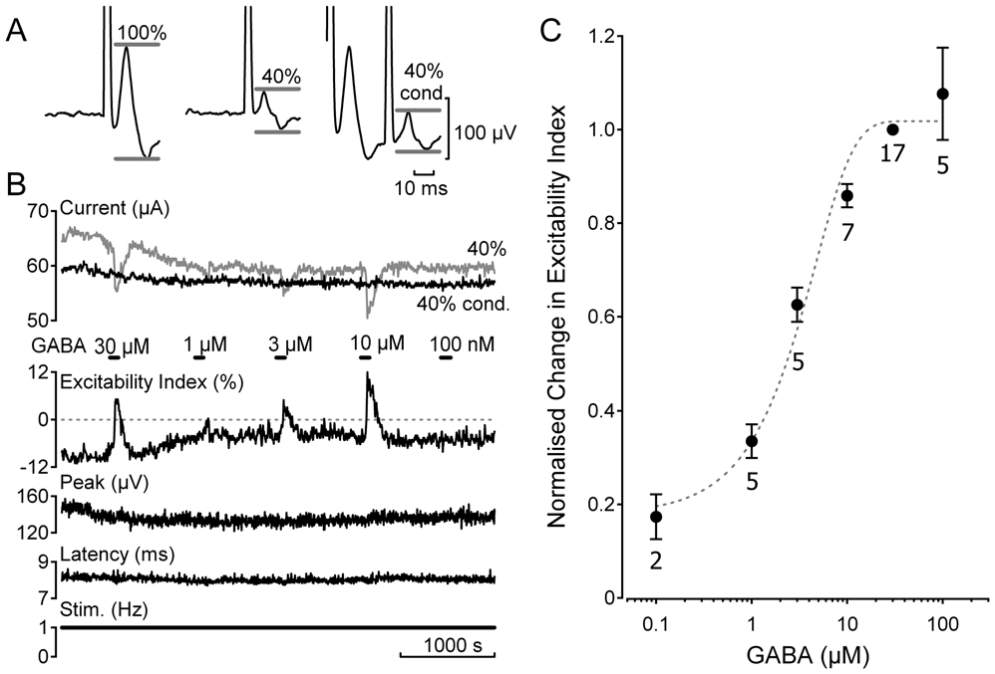
GABA activation of human C-fibres is concentration dependent. The excitability index was determined for C-fibres in human sural nerve fascicles during bath application of GABA (0.1–100 µM). A time window restricted the domain over which the amplitude of the electrically-evoked compound C-fibre action potential was determined (A, grey bars). Excitability index was calculated from the ratio of the current required to evoke an unconditioned C-fibre response of 40% maximum amplitude to that required to evoke a conditioned 40% response, i.e. 30 ms after a supra-maximal conditioning stimulus (40% cond., grey). Negative values of excitability index indicate that more current is required to evoke an unconditioned 40% C-fibre response. Following the addition of GABA (0.1–30 µM, 90 s application) to the bathing solution the excitability index increases, i.e. becomes more positive (B), and the magnitude of this change increases as the concentration of GABA in the perfusing solution increases (B & C). The EC_50_ determined from a sigmoid fit to normalised excitability index on GABA concentration was 6.88±0.01 µM.

In 21 of the 53 (ca. 40%) fascicles examined, bath application of GABA (0.1–100 µM) increased the electrical excitability of C-fibres. An increase in excitability indicates that less current is required to evoke a compound C-fibre response of 40% maximum amplitude. Relative changes in the calculated excitability index (see Methods) reflect changes in membrane potential [26] with an increase in excitability index representing axonal depolarization. GABA (0.1–30 µM) evoked increases in C-fibre electrical excitability are illustrated by example in Figure 1B. GABA produces a concentration dependent increase in the excitability index (Figure 1B & C, upper panel). This increase in excitability index is consistent with GABA depolarizing some of the unmyelinated axons within the fascicle.

**Figure 2:**
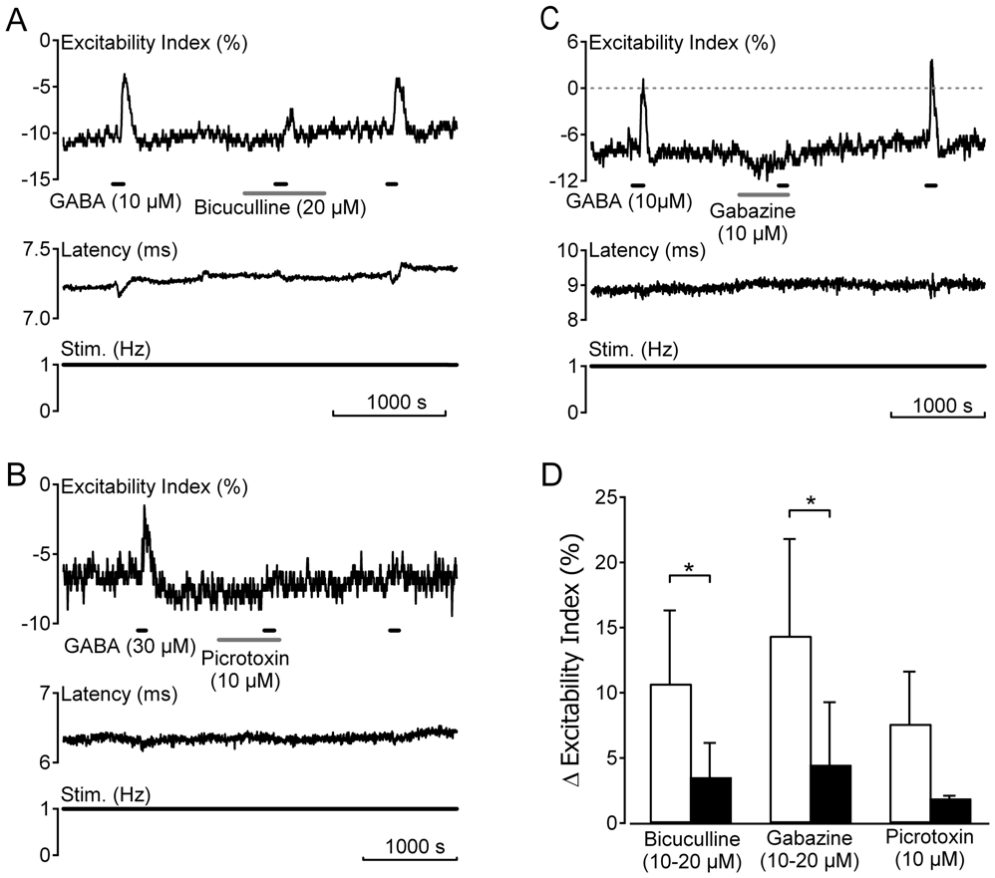
GABA_A_ receptors mediate responses to GABA in human C-fibres. Increases in the electrical excitability index of unmyelinated axons in human sural nerve fascicles following bath application of GABA (10–30 µM) are blocked by prior application of the GABA_A_ receptor antagonists bicuculline (20 µM, grey bar A), picrotoxin (10 µM, grey bar B) and gabazine (10 µM, grey bar C). In contrast to both bicuculline and gabazine, the blocking effect of picrotoxin is not reversed upon wash-out (B). The pooled effect of each compound on the change in excitability index following bath application of GABA (10 µM) is shown in panel D. A significant reduction in the response to GABA (10 µM) was observed in the presence of gabazine (p<0.05, Student's paired t-test) and bicuculline (p<0.05, Student's paired t-test). Owing to the limited availability of human nerve fascicles a statistical comparison was not made for three fascicles exposed to picrotoxin.

**Figure 3:**
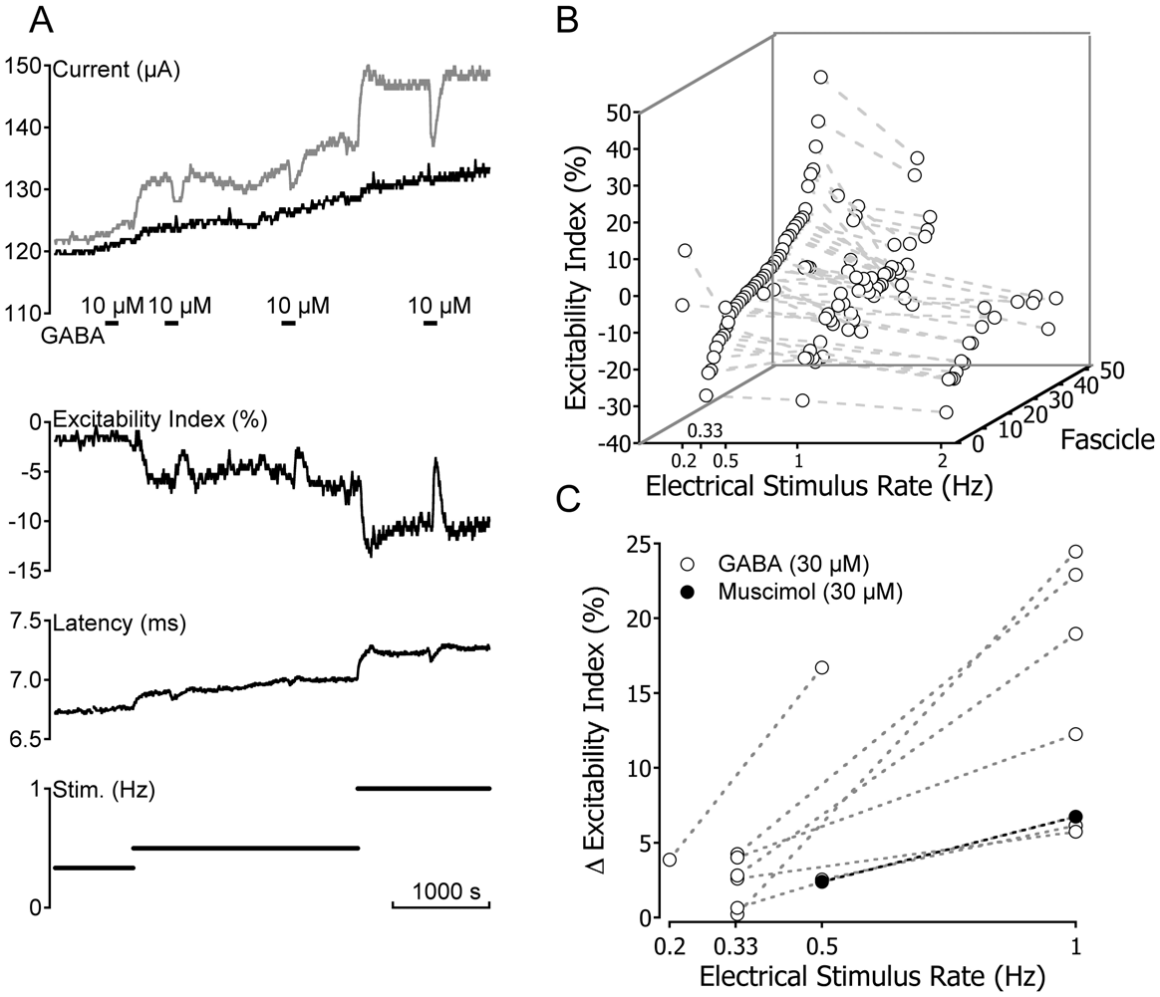
Higher rates of electrical stimulation render human C-fibres less excitable but enhance responses to GABA. The magnitude of the excitability index increase in response to GABA increases with the rate of electrical stimulation. An increase in the rate of electrical stimulation reduces the excitability index of C-fibres (A & B). The absolute magnitude of stimulus rate-induced decreases in excitability index varies (B). The reduction in excitability index produced by increased electrical stimulation rate always increases the magnitude of the change in excitability index observed in response to bath application of GABA or muscimol (10–30 µM, A & C).

The increase in C-fibre excitability index in response to GABA had a rapid-onset, was transient and increased in magnitude in a concentration dependent manner (Figure 1B and C). The EC_50_ of GABA's effect on excitability index was estimated as 6.88±0.01 µM from a sigmoid fit of normalised GABA responses on concentration (Figure 1C). For this analysis, responses to GABA were normalised to the change in excitability index produced by 30 µM GABA. Increases in the excitability index to repeat applications of GABA (10–30 µM for 90 s) at 15 minutes intervals were found to be reproducible and consistent across time. The effect of GABA on peak amplitude and the latency of the compound C-fibre response to supra-maximal electrical stimulation varied. The unmyelinated axons in some fascicles showed no appreciable change in either parameter (0.1–100 µM; Figures 1B, 2B–C & 5B) while in others (see Figures 2A & 3A) the peak amplitude typically increased while the latency decreased.

C-fibre responses to bath applied GABA (10 µM) were mediated by GABA_A_ receptors, being mimicked by muscimol (10 µM, n = 5, data not shown) and substantially reduced by prior application of the GABA_A_ antagonists bicuculline (10–20 µM, reduced to 30.7±7.92%, n = 6, p<0.05 Student's paired t-test), gabazine (10–20 µM, reduced to 29.2±17.14%, n = 7, p<0.01 Student's paired t-test) and picrotoxin (10–30 µM, reduced to 29.56±18.13%, n = 3, Figure 2C). In contrast, 90 s bath application of the GABA_B_ agonist baclofen (100 µM, n = 5, data not shown) was without effect on any of the parameters used to assess the electrical excitability of human C-fibres.

### Relationship between activity-induced and GABA-induced changes in C-fibre excitability

The magnitude of GABA evoked increases in C-fibre excitability index varied within individual fascicles according to the prevailing value of excitability index (see Figure 3). At low rates of stimulation (≤0.33 Hz), absolute values of C-fibre excitability index varied between fascicles (Figure 3B), i.e. C-fibre responses in some fascicles were sub-excitable (index positive) while in others they were super-excitable (index negative). In most fascicles however a decrease in the excitability index of Cfibres can be induced by increasing stimulus rate (Figure 3B, 4 & 5). This in turn increases the magnitude of the change in excitability index produced by bath application of GABA (30 µM, Figure 3A & C). As shown by example in Figure 3A this effect can be considerable. In this example, GABA (10 µM) has no appreciable effect on excitability index at a stimulus rate of 0.33 Hz. However, an increase in the stimulus rate to 0.5 Hz reduced the prevailing excitability index and resulted in a corresponding increase in the magnitude of excitability index change observed in response to GABA (10 µM). Increasing the stimulus rate to 1 Hz, further reduces the prevailing excitability index and thereby increases the magnitude of change in excitability index evoked by GABA (10 µM). In general, the magnitude of the excitability index change produced by GABA (30 µM) or muscimol (30 µM) increases with increasing basal stimulus rate, i.e. as the prevailing excitability index decreases (Figure 3C & 5D).

**Figure 4:**
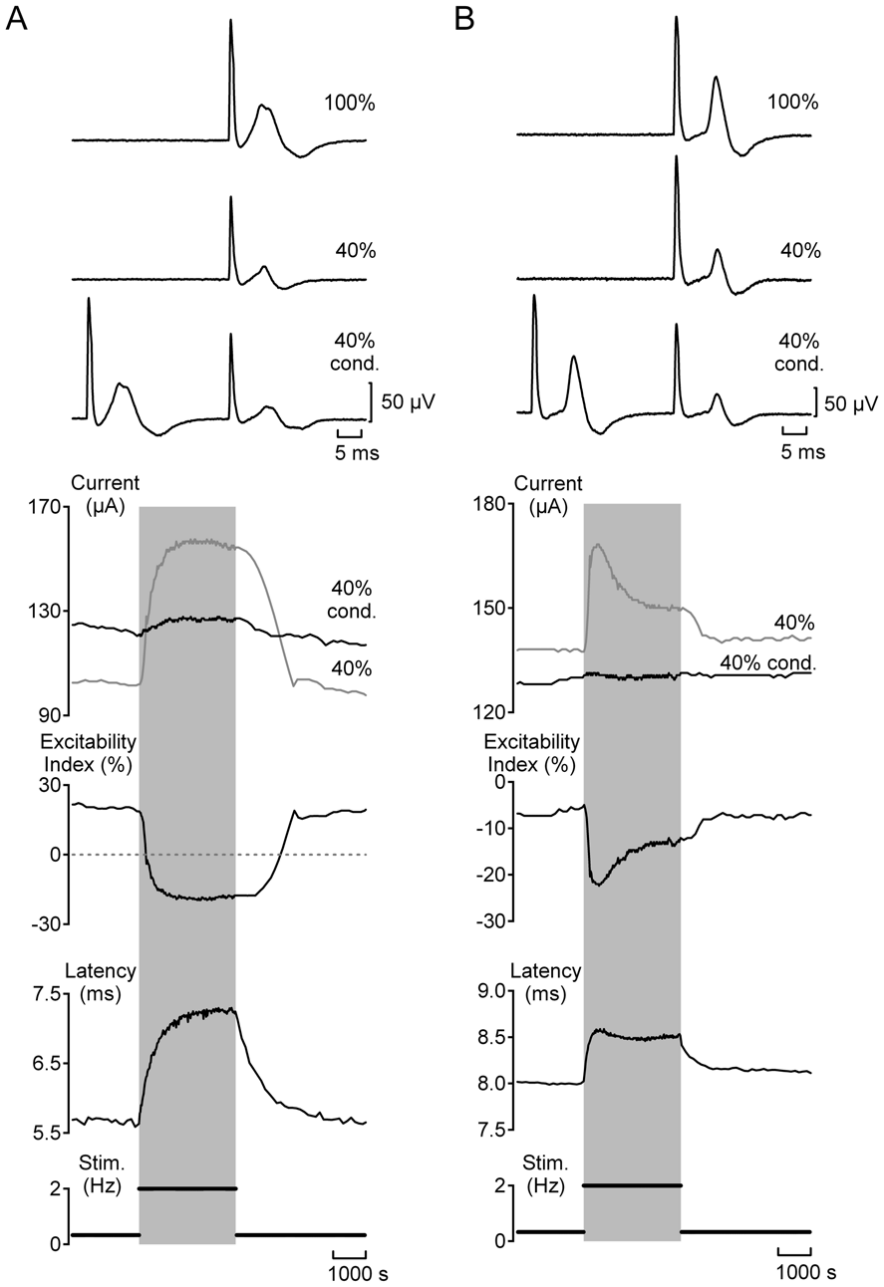
Two types of C-fibre response profile for individual human sural nerve fascicles. Individual fascicles were designated as either Type A (panel A) or Type B (panel B) on the basis of changes in electrical excitability and the latency to half-maximum of the compound C-fibre action potential observed during stimulation at 2 Hz (grey shading). Three features can be used to differentiate the two C-fibre response profiles. Firstly, at low frequencies of stimulation (0.33 Hz), the compound C-fibre response in Type A fascicles is typically sub-excitable (positive excitability index) whereas Type B fascicles are super-excitable (negative excitability index). Secondly, during stimulation at higher frequencies (2 Hz), the compound C-fibre response in Type A fascicles exhibits a monotonic decrease in excitability index and a slowing of conduction latency. For Type B responses, repetitive stimulation initially reduces the excitability index and slows conduction before these changes partially reverse and conduction latency and excitability index both approach a plateau. Finally, during stimulation at 2 Hz, Type A C-fibre responses typically show a reversal from sub- to super-excitability whereas the super-excitability characteristic of Type B responses simply increases in magnitude.

**Figure 5:**
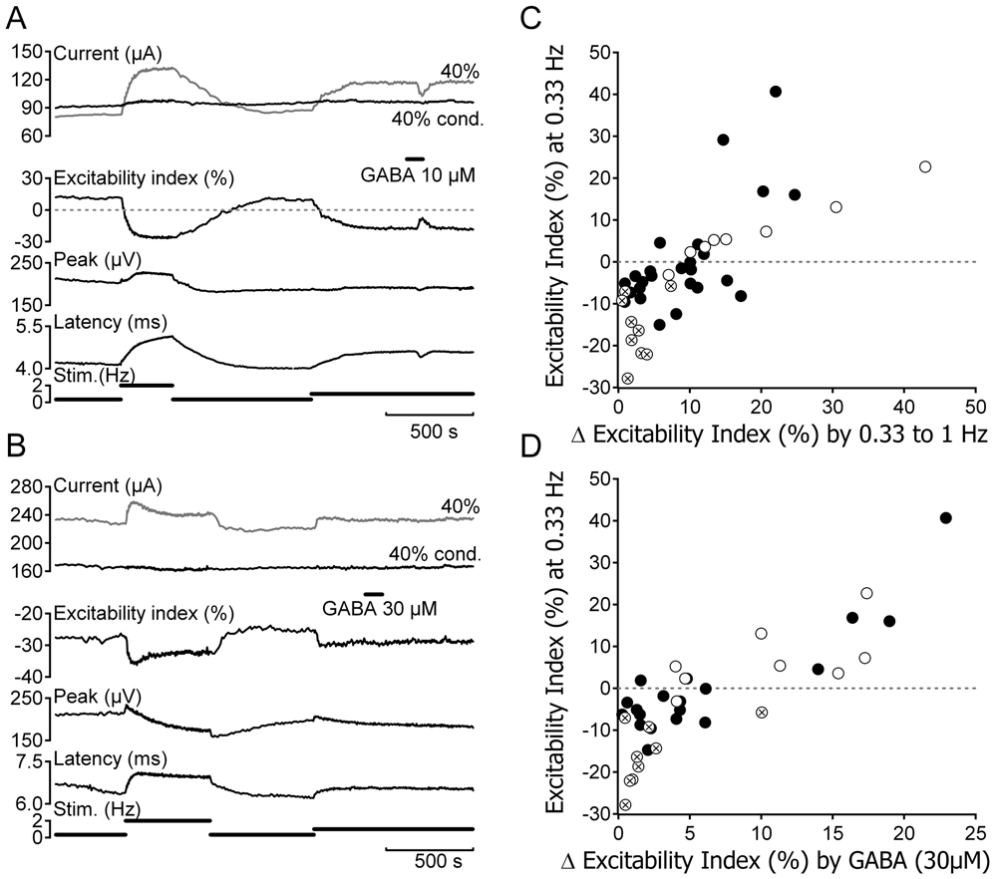
Only a sub-population of human C-fibres respond to GABA. The magnitude of GABA (10–30 µM) evoked increases in excitability index correlate with parameters of electrical excitability. The compound C-fibre response in Type A fascicles (A) is typically sub-excitable (i.e. positive excitability index) at low rates of stimulation and shows a pronounced change in excitability index upon increasing the frequency of repetitive stimulation (open circles, C). In addition, C-fibre responses in Type A fascicles exhibit a large change in excitability index during bath application of GABA (30 µM, open circles, D). In contrast, C-fibre responses electrophysiologically classified as Type B (B) are typically super-excitable at low stimulus frequencies, show a modest change in excitability upon repetitive stimulation at 2 Hz (encircled crosses, C) and typically respond poorly or not at all to GABA (30 µM, encircled crosses, D). The filled markers in panels C and D represent fascicles for which a classification based upon the C-fibre response profile to repetitive electrical stimulation at 2 Hz was not determined.

### GABA increases axonal excitability in a subset of human unmyelinated axons

Unmyelinated axons in twenty-one of the 53 nerve fascicles exhibited a change in electrical excitability index in the presence of GABA (10–30 µM). This restriction of GABA sensitivity to a subset of fascicles may reflect a selective expression of GABA_A_ receptors in a sub-population of human unmyelinated axons. To examine this premise an attempt was made to determine whether a particular axonal sub-type was sensitive to GABA. The global behaviour of C-fibres within individual nerve fascicles was therefore determined according to their excitability index at 0.33 Hz and the profile of change in excitability index and response latency exhibited during stimulation at 2 Hz (see Figure 4).

Seventeen fascicles from five nerves (each taken at biopsy) were subject to the electrical stimulus protocol and two general response patterns were observed. We have designated the two response profiles Type A and Type B and the incidence of response profile subtype in fascicles arising from individual nerves is summarized in Table 1. Nine of the 17 fascicles contained Cfibres with a global Type B profile. During stimulation at 2 Hz this profile comprised an initial increase in the latency to half-maximum of the compound C-fibre response that subsequently reversed, i.e. the velocity of conduction in C-fibres initially decreased before increasing slightly and approaching steady-state (Figure 4B & 5B). At low stimulus rates (<0.33 Hz) the Type B response profile was super-excitable (negative excitability index, Figure 5C) and during stimulation at 2 Hz became more super-excitable (Figure 4B & 5B). Only one of the nine Type B fascicles showed an appreciable increase in C-fibre excitability index in response to bath application of GABA (30 µM, Figure 5D). C-fibre responses in eight fascicles had a Type A profile indicating that during stimulation at 2 Hz a monotonic slowing of latency and a parallel decrease in excitability index were observed (Figure 4A & 5A). C-fibre responses in seven of the eight Type A fascicles were sub-excitable (positive excitability index, Figure 5C) at low stimulus frequencies but became super-excitable at higher stimulus rates (Figure 4A & 5A). The excitability index of C-fibres in all eight Type A fascicles increased following bath application of GABA (30 µM, Figure 5A, B & D). For individual nerve fascicles the excitability index of C-fibres determined at 0.33 Hz ranged from −27.9% to 40.7% and was predictive for the absolute magnitude of the excitability index change upon increasing the rate of electrical stimulation from 0.33 Hz to 1 Hz (Figure 5C) as well as for the magnitude of the change in excitability index observed in response to bath application of GABA (30 µM, Figure 5D).

**Table 1:** Incidence of C-fibre responses by sub-type in human sural nerve.

Nerve ID	Total fascicles	Type A	Type B
AR 180	3	2 (2)	1 (1)
KF 182	5	1 (1)	4 (0)
WF 183	2	0	2 (0)
IW 184	4	4 (4)	0
MH 185	3	1 (1)	2 (0)

The collective population of unmyelinated axons within individual nerve fascicles was characterized by a period of electrical stimulation at 2 Hz (see Figure 4). C-fibre responses were classified as either Type A or B (see Results). The incidence of each type of response is shown as a function of the nerve of origin (Nerve ID). The values in parentheses indicate the incidence of C-fibre responses to GABA (30 µM).

## Discussion

The application of conventional intracellular electrophysiological recording techniques to unmyelinated axons in peripheral nerves is precluded by their small size. Accordingly the effects of GABA on C-fibres in single fascicles of human sural nerve can only be examined indirectly. We have used electrical threshold tracking [27] to determine an excitability index that serves as an indirect means of assessing relative changes in membrane potential [26]. Exposure of human sural nerve segments to GABA (1–100 µM) increased the excitability index of a largely nociceptive (see below) population of unmyelinated axons via GABA_A_ receptor activation. The increase in electrical excitability of human unmyelinated axons in response to GABA is consistent with axonal depolarization and suggests that human unmyelinated axons have an elevated intracellular chloride concentration. The findings indicate further that unmyelinated axons in peripheral human nerve exhibit a selective chemosensitivity. The demonstration of functional GABA_A_ responses in human unmyelinated axons supplements the body of work showing that GABA_A_ receptors are functionally expressed in axons in the mammalian peripheral and central nervous system [1], [13], [28] and may have implications for the pathogenesis of some forms of neuropathy.

The increase in human C-fibre electrical excitability seen in response to GABA was mediated by GABA_A_ receptors. The effect of GABA was blocked by bicuculline, picrotoxin, and gabazine (Figure 2). While this profile is consistent with that observed for GABA_A_ mediated currents in isolated human embryonic DRG neurones [5], it contrasts with GABA responses evoked in cultured human adult DRG neurones which are insensitive to both bicuculline and picrotoxin [29]. The basis of this apparent discrepancy is not clear. It may reflect differences in GABA receptor subunit composition or may be due to differences in sampling between unmyelinated axons and a heterogeneous population of DRG somata with potentially either myelinated or unmyelinated axons. To what extent, if any, an underlying neuropathology may contribute to this discrepancy between samples of human sural nerve taken at biopsy and isolated human DRG-somata is also not known. Baclofen did not affect the excitability of peripheral human axons which is consistent with reports from isolated human DRG neurones [4]. However both GABA_A_ and GABA_B_ receptors have been immunohistochemically identified in cultured Schwann cells from rat [30] and segments of rat sciatic nerve [31] and their activation by GABA agonists has been suggested to influence the expression of myelin proteins P0 and PMP22 [30].

Changes in the electrical threshold of human C-fibres in response to GABA are thought to reflect axonal depolarization. This view is supported by previous evidence from rat axons [11] as well as the observation that rat sympathetic [32] and frog DRG [14] neurones have an elevated intracellular chloride activity, a feature likely to extend to primary sensory neurones in mammals. Consistent with this idea is the observation that GABA evokes an inward depolarizing current in isolated human DRG neurones [5]. GABA also increases the excitability index determined in unmyelinated axons traversing human nerve fascicles (Figure 5) and a similar increase in the excitability index of C-fibres can be induced with depolarizing current applied extracellularly to human nerve fascicles [33]. Further support for the notion that GABA depolarises unmyelinated axons is the observation that C-fibre responses to GABA increase with stimulus rate (Figure 3). Peripheral [34] as well as central [35] unmyelinated axons hyperpolarize upon repetitive activation, an effect attributed to an increase in Na-K-ATPase activity [36]. Activity-induced hyperpolarization in unmyelinated axons would be expected to increase the difference between membrane potential and the reversal potential of the GABA_A_ receptor, itself dominated by E_Cl_, and thereby enhance the magnitude of GABA-mediated depolarization of peripheral unmyelinated axons.

C-fibre responses to GABA were restricted to a subset of human nerve fascicles which is consistent with the notion that only a subpopulation of human unmyelinated axons expresses functional GABA_A_ receptors. Indeed, immunohistochemical labelling of cat skin indicates that β2/β3 and α1 GABA_A_ receptor subunits are restricted to approximately 12% of unmyelinated axons [37]. Following initial observations in single axons in rat [38], it has also been possible to correlate the receptive class of individual unmyelinated axons in humans on the basis of the change in axonal conduction velocity they exhibit during a period of stimulation at 2 Hz [39]. In particular, when normalised for differences in their basal conduction velocity, axons from nociceptors exhibit a more pronounced slowing of axonal conduction velocity than do non-nociceptive sensory afferents [39], [40] and sympathetic efferents [41] during repetitive activation. We show here that compound C-fibre action potential responses in isolated human nerve fascicles fall broadly into two populations on the basis of the changes in conduction latency and excitability they exhibit during stimulation at 2 Hz (Figure 4). This result was somewhat surprising given that human microneurographic studies suggest a somatotopic rather than a functional grouping of unmyelinated axons [42] and that human sural nerve fascicles may contain upward of one thousand unmyelinated axons [43] within which even individual Remak bundles may contain heterogeneous axonal sub-types [44]. Nevertheless, based on functional characteristics, it appears that the characteristic compound electrical C-fibre response profile of individual human nerve fascicles is largely consistent with either nociceptive or non-nociceptive unmyelinated axons (Figure 4). For instance, fascicles classified as Type A exhibited properties consistent with their containing predominantly nociceptive unmyelinated axons, they were sub-excitable at low stimulus rates and showed pronounced activity-dependent slowing. Moreover, the C-fibre excitability index determined in all eight Type A increased in response to GABA (Figure 5D). In contrast, only one of the nine Type B fascicles deemed to be comprised largely of non-nociceptive unmyelinated axons responded to GABA (Figure 5D). In rat sciatic nerve, GABA responses in myelinated nerve fibres are also restricted to sensory axons, motor axons do not respond to GABA [13], [45]. In this context, while GABA has been shown to modify formalin-induced behavioural responses in cats [37] it would be interesting to examine which sensations, if any, GABA might evoke in human subjects.

Functionally, the activation of axonal GABA_A_ receptors has been associated with a reduction in the safety factor of action potential initiation [19] and conduction [46] as well as the regulation of neurotransmitter release [47]. Verdier *et al.* (2003) propose that axo-axonic GABAergic synapses exert an inhibitory effect on axonal discharge such that tonic activity is periodically interrupted resulting in a bursting pattern. This is consistent with the dependence of GABA's efficacy upon preceding activity observed here in human C-fibres (Figure 3) and suggests that GABA may exert a more pronounced action on active C-fibres. Another possible role of axonal GABA_A_ receptors involves the increase in the intracellular chloride concentration of DRG neurones observed in response to both inflammatory mediators [23] and following peripheral nerve injury [24]. Speculatively, under such conditions, it is possible that GABA_A_ receptor activation produces exaggerated depolarizing responses that may acutely initiate action potentials or open voltage-gated Ca^2+^ channels that are present in unmyelinated human axons [48].

The data establishes the functional expression of GABA_A_ receptors in the axonal membrane of a sub-population of unmyelinated afferent human nerve fibres that most probably comprises nociceptors. It has been postulated that a loss of interneuron mediated GABAergic inhibition within the spinal dorsal horn may contribute to the establishment and maintenance of some neuropathic pain states (see [49], [50]). Indeed current therapeutic strategies currently being developed in mice are aimed at reversing this GABAergic disinhibition through sub-type selective activation of GABA_A_ receptors expressed in the spinal dorsal horn [50]. If the functional GABA_A_ receptor expression in peripheral human unmyelinated axons extends further to the centrally projecting axons of sensory neurones, this observation provides considerable impetus for the translation of GABA_A_ receptor targeted strategies to humans.

## Materials and Methods

### Ethics Statement

Approval for the experimental use of human tissue was granted by the Ethics Committee of the Medical Faculty of the University of Munich (Project Number 348/00). Patients were informed about the biopsy procedure by an anaesthetist one day prior to surgery at which time the patient's written consent to the removal of an additional portion of nerve for research purposes was established.

Experiments were carried out on isolated fascicles of human sural nerve obtained from 16 patients (9 male, 7 female) previously scheduled for either sural nerve biopsy or lower limb amputation. Segments of sural nerve were obtained from patients ranging in age from 20 to 89 years with a median age of 68 years. The underlying diagnosis precipitating biopsy was either polyneuropathy of unknown aetiology or peripheral artery occlusive disease. Neither the profiles of electrical excitability nor the pharmacological responses of C-fibres within individual fascicles were correlated with the prevailing pathological classification.

Segments of human sural nerve obtained at biopsy were typically 15–25 mm long. Individual nerve fascicles were carefully extracted from isolated segments of sural nerve by gently pulling them free with jeweller's forceps. Isolated fascicles were mounted between suction electrodes in an organ bath. Each end of the nerve fascicle was drawn into a glass suction electrode and embedded in Vaseline to establish both a mechanical and a high resistance electrical seal. The organ bath (volume ca. 1 ml) was perfused continuously at a rate of 6–8 ml.min^−1^ with physiological solution of the following composition (in mM) NaCl 117, KCl 3.6, CaCl_2_ 2.5, MgCl_2_ 1.2, D-glucose 11.0, NaHCO_3_ 25, NaH_2_PO_4_ 1.2, bubbled with 95% O_2_/5% CO_2_ to pH 7.4. The temperature of the solution perfusing the bath was held constant at 34°C.

Axonal excitability was determined in C-fibres by stimulating the nerve fascicle electrically with constant current pulses (A395, WPI, Sarasota, USA). A silver wire inside the stimulating suction electrode served as the anode and a second silver wire wound around the suction pipette in the organ bath served as the cathode. Extracellular signals were recorded over the sealing resistance of the second suction electrode with a differential amplifier (NPI, Tamm, Germany). The distance between stimulating and recording electrodes was typically 4–8 mm. A window discriminator allowed the C-fibre response to be monitored in isolation.

An electrical stimulus protocol was used to examine the effect of GABA on the excitability of C-fibres in sural nerve fascicles. Constant current pulses of fixed duration (1 ms) and varying amplitude were used to track changes in C-fibre excitability with QTRAC software (© Institute of Neurology, London, UK). Three interleaved stimulus parameters were monitored sequentially. Firstly, a supra-maximal current intensity was established at which the amplitude of the compound C-fibre response was maximal and this was designated a 100% response (100%, Figure 1A). Electrical threshold was the second stimulus parameter monitored. This is defined as the current required to elicit a compound C-fibre response with an amplitude 40% that of the response to supra-maximal stimulation (40%, Figure 1A). The stimulus current required to evoke a 40% amplitude C-fibre compound action potential was continuously adjusted by the QTRAC software. The third parameter was post-spike electrical threshold and this is the stimulus current required to maintain a conditioned C-fibre compound action potential response of 40% amplitude, that is 30 ms after a conditioning supra-maximal electrical stimulus (40% cond., Figure 1A). The difference between the conditioned and unconditioned current intensities normalised to the stimulus intensity of the conditioned response is defined as the ‘excitability index’ and is expressed as a percentage.

Gabazine (Biotrend, Cologne, Germany), baclofen, bicuculline, gamma-aminobutyric acid, muscimol and picrotoxin (Sigma, USA) were stored as stock solutions in distilled water. The desired concentration of each substance was achieved by dilution from stock into the solution perfusing the bath on the day of each experiment.

Data are expressed as mean and standard deviation for comparisons between groups while for population descriptors mean and standard error of the mean are indicated. Student's t-test was used for statistical comparisons of paired datasets. Curve fitting was performed in Igor Pro (Wavemetrics, Lake Oswego, USA) which uses the Levenberg-Marquadt algorithm for least-squares minimization.
